# Places in Information Science

**DOI:** 10.1002/asi.24194

**Published:** 2019-03-12

**Authors:** Ross S. Purves, Stephan Winter, Werner Kuhn

**Affiliations:** ^1^ Department of Geography University of Zurich Zurich Switzerland; ^2^ University Research Priority Programme, Language and Space University of Zurich Zurich Switzerland; ^3^ Department of Infrastructure Engineering The University of Melbourne Melbourne Australia; ^4^ Department of Geography and Center for Spatial Studies University of California Santa Barbara California USA

## Abstract

Human spatial concepts, such as the concept of *place*, are not immediately translatable to the geometric foundations of spatial databases and information systems developed over the past 50 years. These systems typically rest on the concepts of objects and fields, both bound to coordinates, as two general paradigms of geographic representation. The match between notions of place occurring in everyday *where* questions and the data available to answer such questions is unclear and hinders progress in place‐based information systems. This is particularly true in novel application areas such as the Digital Humanities or speech‐based human–computer interaction, but also for location‐based services. Although this shortcoming has been observed before, we approach the challenges of relating places to information system representations with a fresh view, based on a set of core concepts of spatial information. These concepts have been proposed in information science with the intent of serving human–machine spatial question asking and answering. Clarifying the relationship of the notion of place to these concepts is a significant step toward geographically intelligent systems. The main result of the article is a demonstration that the notion of place fits existing concepts of spatial information, when these are adequately exploited and combined.

## Introduction

Places are referred to in news items, travel guides, websites, social media, and captions of photographs, as well as in verbal communication, search queries, or speech input to navigation systems and location‐based services. In written or spoken form, natural language uses place names and descriptions as references to locations that are meaningful in the context of a conversation. Places are often the subject of shared implicit knowledge between speaker and recipient, with sufficient agreement for communication to succeed.

Conceptually, place information is of particular importance in human geography (for example, Cresswell, 2004; Tuan, 1977), and increasingly so—with the rise of systems relying on natural language for interaction—in information science and information systems. However, despite some early discussion of place in an information science context (for example, Harrison & Dourish 1996) the notion of place has remained peripheral in discussions of spatial information in information science. We address this omission here, using a theory to bridge the gap between notions of place and information science, and we suggest how information science and information systems might better cope with place information.

The study of geographic information and the foundations of geographic information systems (GIS) are concerned with the conceptualization, capture, management, and analysis of geographic information (Duckham, Goodchild, & Worboys, 2003; Goodchild, 1992; Longley, Goodchild, Maguire, & Rhind, 2015). GIS typically represent geographic environments in one of two general ways (Couclelis, 1992; Goodchild, Yuan, & Cova, 2007):

- *object*‐based: representations of features of the geographic environment using geometric objects with normally crisp boundaries and associated attributes;
- *field*‐based: space‐filling representations of properties of the geographic environment, using a regular or irregular sampling of attributes.

Despite many discussions about more nuanced models, such as vaguely defined geographic objects and their possible representations (for example, Burrough & Frank, 1996), these simpler models continue to dominate GIS and other spatial information systems. This is perhaps unsurprising, as they have proved well‐suited to many tasks related to spatial data, especially where such data represent either administrative or environmental phenomena. However, they fall short of representing the intuitive geographic concept of place, which plays a key role in everyday human experience and communication. This shortcoming complicates human–computer interaction and, among other tasks, the integration of crowd‐sourced (a.k.a. volunteered) geographic information with authoritative sources.

Many current information systems deal with places in an ad‐hoc and impoverished manner, as points of interest (POI), typically with no extent and with a bias toward commercial entities. Most toponym databases and gazetteers (Goodchild & Hill, 2008) simply store place *names* and alternates, place *types* in some more or less ad‐hoc taxonomy, and place *geo‐references* to points or occasionally polygons in a coordinate system. Although they have the capacity to link informal, place‐based human discourse with formal, coordinate‐based systems, they are too limited in themselves to capture the richness of place information. They only deal with the place types listed in their taxonomies (typically some geographical features, such as mountains or lakes, and administrative units) and they do not represent relationships other than the part‐of‐relationships of hierarchical administrative subdivisions. They fail to account for proximity and adjacency of places, and they allow for computing nonsensical distances between places and their parts (for example, the distance between London and Buckingham Palace). Other kinds of spatial information systems admit even less place semantics. For instance, features in OpenStreetMap may lack names and be defined only by geometry, despite having some “placeness.” As Elwood, Goodchild, and Sui (2013) pointed out, place is also absent from many taxonomies of spatial databases and information systems. The framework data types developed by the US Federal Geographic Data Committee,^1^ for example, cover a set of basic data themes considered central to GIS usage by many government and associated organizations, including geodetic control, cadastre, ortho‐imagery, elevation, hydrography, administrative units, and transportation. These themes are typical of such efforts, in that they focus on physical objects and properties (for example, rivers, roads, or heights) and administrative units (for example, county boundaries) that have been the subject of traditional mapping, but leave little room for place concepts that are vague or contested.

We are certainly not the first researchers to suggest fuller recognition of place in information science and more adequate representations in information systems. Egenhofer and Mark called for what they termed *Naïve Geography*—a set of formal models in information systems that “captures and reflects the way people think and reason about geographic space and time” (1995, p. 4). Golledge and Stimson suggested that “regardless of whether the tangible or intangible position was taken with respect to examining the sense of place, it should be possible to develop either a subjective or an objective scale (or some combination of the two) that captures the essence of a place” (1997, p. 417). Sui and Goodchild (2011, p. 1744), theorizing about GIS as media, observe “a new level of urgency for theoretical works to reconcile the world of space (traditional GIS) and the world of place (social media).” Acknowledging the rise of volunteered geographic information (VGI), Elwood et al. observe: “VGI research invites a more place‐centric perspective and may even stimulate the development of a parallel, platial geographic information system” (2013, p. 362). Roche called places “key operators for digital spatiality” (2014, p. 709).

Yet the translation of human spatial concepts into information system representations has proven surprisingly hard. This is, we argue, mainly due to the different ways of expressing spatial knowledge in geometries and in words, the role of place as a social construct (Goodchild & Li, 2012), and the influence of context. Note that the challenge is *not* to find data models for places; once it has been established what needs to be represented about places, data models will be available. These may include vector and raster data models of locations, as well as graph data models and others to represent thematic, temporal, and additional spatial aspects. Rather, the challenge we address from a theoretical standpoint is *semantic*: What is place information *about*?

Some researchers have asked whether the concept of place may be “simply too vague to be formalized, except in very narrowly defined circumstances” (Goodchild, 2011, p. 22). We disagree and posit that place information can be captured at any desired levels of vagueness or richness through existing concepts and theory in information science. Our approach, based on a recently proposed set of core concepts of spatial information (Kuhn, 2012), bridges the gap between the vague, flexible, and socially constructed concept of place and the formalisms of information science. The core concepts were developed as part of a more general effort at theory building for transdisciplinary research relying on spatial concepts. At their heart lies a desire to develop a small set of high‐level concepts that allow domain specialists to express questions without detailed knowledge of GIS as a technology. We adopted the core concepts for this article because we believe that decoupling of place from GIS data models through the core concepts will allow a wide range of domains to model place in their own way. We demonstrate that our proposal is not only powerful enough to address the requirements articulated in the literature on place and the shortcomings in information system implementations, but also simple enough to be practically applicable and useful.

The remaining sections of this article present this approach from three perspectives. First, our approach is introduced, embedded in the core concepts, and its characteristics are explained. Then we show how it is capable of accounting for the dimensions of place identified in the literature. Finally, we demonstrate how it supports dealing with places in information systems through a series of examples.

## Place and the Core Concepts of Spatial Information

Having observed the limitations of standard conceptualizations of geographic environments when it comes to place modeling, we adopt a richer and more recent ontology of geographic information and show how it is used in conceptualizing place. Because we will also demonstrate later how this choice supports computation, in addition to representation, the chosen ontology must include computation.

We chose Kuhn's ontology of core concepts of spatial information (Allen et al., 2016; Kuhn, 2012) which, in its latest form, includes a base concept *location,* four content concepts: *field*, *object*, *network,* and *event*, and three information quality concepts: *granularity, accuracy,* and *provenance*. For the purpose of this article, the quality concepts are out of scope. Each of the base and content concepts describes a phenomenon in space *and* time, and comes with a small set of core computations on its instances:

- *Location* is the base concept of all spatial information, enabling *where* questions. The concept comes in two commonly used forms, location as a spatial relation between what is located and what locates it; and location as a region in the world defined by such relations. For example, stating the relation that “the bus station is near the church” defines the region “near the church” as a location. Similarly, giving geographic coordinates for a town defines a position as the town's location, and a statement like “I'm on my way home” defines a path location. Locations have no identity and are immutable in time. Although different locations can be ascribed to a place over time, the locations themselves are immutable parts of space. Locations can be defined in any space, including virtual spaces, and for any number of dimensions (between zero and three for geographic spaces). Positions are atomic regions, that is, the smallest elements of space; they may be points described by coordinates (in vector geometry) or cells described by a set of indices (in raster geometries). Computations on locations include distances and directions between them as well as lengths and areas, position‐in‐location queries (for example, point in polygon), and reference system transformations (for example, from geographic to plane coordinates).
- A *field* is a function that returns an attribute value for any position and time in its domain. Examples of phenomena conceptualized as fields include temperature, interpolated over a region or heights attributed to pixels in a grid. Core computations on fields return selected attribute values at a given position, or modify and combine fields using functions applied across the field (for example, calculated slope on an elevation field).
- An *object* is a uniquely identifiable entity existing in space and time and having well‐defined properties as well as relations with other objects. Examples of objects include buildings, mountains, and people. A geographic object is always located. Contrary to the traditional GIS object notion (rooted in the process of map digitization), the object core concept does not require a boundary, although objects are always bounded (that is, confined within a finite region, the spatial equivalent of a bounded set). This object concept blends the ideas of crisply bounded objects (for example, buildings) with that of features of a surface (for example, a mountain as a feature of the earth's surface), which often cannot be assigned a boundary. Objects can be generated from fields (for example, by identifying regions in a field whose attribute values satisfy some constraints), as well as from other objects (for example, by aggregating or subdividing them). Core computations on objects primarily return property values and objects satisfying certain relations (spatial, temporal, and/or thematic).
- A *network* is a set of objects in space and time (forming its nodes) connected by a binary relation (forming its edges, which may be time‐dependent). The edges can be reified into objects (for example, roads between cities, or utility lines), or they can remain abstract (for example, social connections). Core computations on networks answer, for example, questions about the reachability and centrality of nodes, or about the shortest paths in networks.
- An *event* is anything that happens within a bounded space and time. Geographic examples include earthquakes, storms, and traffic jams. Note that although events are primarily temporal (as well as played out in space), all core concepts have in fact temporal aspects (as the short descriptions above indicate). Events account for changes in their participants. For example, a storm changes temperatures and may move objects or block network connections. Event participants are instances of fields, objects, and networks. Core computations on events answer questions about event durations, the temporal order of events, event participants, and possible causes or consequences of events.

How can we conceptualize places in these five core concepts? According to Vasardani and Winter (2016, p. 243), a place is “a location (in an environment, not in an empty space) with properties that give it ‘shape and character’ and which enable conversations about place.” We add here that places need not be permanent, and may be ephemeral or recurrent; thus, time is inherent to any definition of places. This characterization suggests that a place turns a location into an object with properties and a shared identity. Because locations are treated as values in the core concepts, without identity, they cannot have properties that change, and cannot be referred to as places in conversations. However, any location can become an object by giving it a shareable identity, allowing it to have properties and relations. For example, the location examples given above are either thought of as objects already (the town represented by a position) or can be turned into place objects (the region near the church, my way home).

Thus, a straightforward derivation of place from the core concepts is:

A *place* is an object resulting from a shared identification of a location. As an object, it may become a part of a network and participate in events.

Note that this derivation expresses an ontological commitment, not a definition. By stating an ontological commitment, which establishes a *language* to talk about places, we refrain from defining place in a single normative way. Instead, our ontological commitment admits any place definitions, as long as they fulfill the following three conditions:

1. Places must be identifiable through some set of definable properties and/or relationships, and are thus unique geographic objects.
2. Because places are geographic objects they must have locations.
3. The identity of a place must be shared—emphasizing that places are social constructions and emerge from some form of human consensus.

Our approach enables formalizations of place based on the core concepts, and admits a variety of equally valid definitions of place. By expressing an ontological commitment, rather than a definition, we move away from previous, largely unsatisfactory attempts at defining place, and replace it by a number of possibilities and conditions for place definitions. Thus, a definition focusing on human experience and activities (for example, places where people gather to enjoy themselves) is just as valid as one that identifies places emerging from networks of trading relations in the 19^th^ century. Note, however, that a place definition must fulfill all three conditions: an arbitrary set of coordinates is not a place, neither is a single tree in the forest. But if that tree can be identified, and if its identification is shared, through a name or some set of properties, then the tree becomes a place. In the following, we explore in more detail how the core concepts help us to understand (and thus model) place conceptually.

The location concept allows places to be defined by arbitrary spatial relations, geometrically or linguistically, allowing us to depart from the geometrically anchored models of current GIS. Thus, the center of a town, the north flank of a mountain, or the midpoint of a journey can all be locations that are experienced and referred to as places. Our derivation of places from locations by giving them identity allows for places to be any meaningful locations, real or imagined, measured or described, as long as they offer some sharable experiences.

A place's location can be derived from a field, based on characteristic attribute values. For example, Southern California can be located by a field measuring the strength of people's agreement that elements of geographic space belong to it (Gao et al., 2017). The derivation, furthermore, implies that places result from locations by *naming* (downtown Santa Barbara) or description (“the place where we met”). The names and descriptions use the shared identity of a place in a community to refer to it. Naming places, in turn, enables recursive locating. For example, locating a building near downtown Santa Barbara refers to downtown Santa Barbara and applies the spatial relation near to it, defining a new location. The latter location is, as such, not (yet) a place, whereas the former (downtown Santa Barbara) clearly is, because it embodies, for some community, some shared notions about this location.

Naming or describing places can refer to different locations over time or to a location that cannot be observed anymore; consider Poland, which has changed its location dramatically over time, or a corner shop from your childhood, which may have disappeared, but still be recognized as a place, suffused with meaning, by you and your childhood friends. In turn, different places may emerge at the same locations because nothing excludes the existence of multiple, fully, or partially overlapping places in both space and time.

This naming or describing turns locations into objects. All of the aspects that make place a richer notion than simply a location can be accounted for by properties and relations attributed to places as objects. For example, the corner shop may be referred to in contexts where properties other than its location are in focus. “The shop has an area of 100m^2^” treats it as real estate, or “the shop closed in 1985” gives historical context.

Seen as objects, places can then also become nodes or edges in networks (Vasardani, Timpf, Winter, & Tomko, 2013). For example, a town is a place with transportation links to other places, and a bridge crossing a river between two countries can become a place where a prisoner exchange occurs. In a network, places may be located qualitatively, through network topology, without recourse to coordinates. Qualitative notions of place, regardless of possible geometric models, have become widely accepted in artificial intelligence (Kuipers, 1978; Kuipers & Byun, 1991). They are perception‐based and typically identified as local maxima of some distinctiveness measures (Kortenkamp & Weymouth, 1994; Tapus, 2005). Place graphs represent places as nodes, connected by edges if, for example, one place can be reached directly from another.

Places can also participate in events (Chan, Vasardani, & Winter, 2014). For example, in “the café is popular for weddings” an event at the place is in the foreground. This link to events underlies suggestions to describe places by what they afford, that is, by what they offer to human beings (Gibson, 1979; Jordan, Raubal, Gartrell, & Egenhofer, 1998; von Uexküll, 1980). We do not suggest, however, that events and places are the same—rather, places may host or participate in events (as in the above example) and may emerge from events (think for instance of a historic battlefield).

With this section we have embedded the notion of place in the theory‐based, formally specified core concepts of spatial information. We have constructed place from the core concepts of location and object, and we linked it to the field, network, and event concepts. Equipped with this simple yet powerful understanding of place, we show next how it is capable of accounting for the dimensions of place identified in the literature.

## Accounting for the Cognitive Dimensions of Place

The distinction between place, as an object having a location with shared identity, and location itself in the prior section has parallels to the often‐discussed duality of space and place (for example, Agnew, 2011; Tuan, 1977). Tuan, for example, argues that place is linked to experience, whereas space is not. Agnew (1987, 2011) goes a step further and identifies three dimensions of place: a location (somewhere in space, where things happen and which can be related to other locations), a locale (“the where of social life and environmental transformation”), and a sense of place (“identification with a place as a unique community, landscape and moral order”). We show here that Agnew's three dimensions can be fully captured by a place conceptualization in terms of objects resulting from shared references to locations, that is, that they are compatible with the approach to define place within core concepts. Agnew also argued for thinking of places in relation to other places, and not as “bounded, isolated entities,” thus supporting the entailment of our derivation that places as objects do not necessarily have a boundary and that they can form networks and participate in events. Finally, Agnew recognized that places might be mobile (for example, a train) or even virtual (for example, an Internet chat room), and need not be permanent.

Interestingly, Agnew's model has striking parallels with Shatford's (1986) seminal work on image classification, and in particular mirrors the *specific*, *generic,* and *about* aspects of the *where* facet in her model. Thus, in natural language, location is typically communicated through references to places, such as place names (toponyms; “London”) or place‐identifying count nouns (definite object types; “the bar”). These two roles have, as specific or generic elements of the *where* facet (Shatford, 1986), been demonstrated to serve different purposes in information classification and search (Armitage & Enser, 1997). To serve the purpose of localizing other objects in locative expressions they must themselves be localized (Scheider & Janowicz, 2014; Vasardani et al., 2013). As proper names, toponyms are implicitly specific referring expressions with no guaranteed semantic meaning (Coates, 2006), and are subject to ambiguity (Smith & Crane, 2001). Thus, reasoning about their relationships to other locations typically requires both identification of an unambiguous referent and knowledge about their type (Leidner, 2007), which may be provided by the conversational context (emphasizing the shared identification). “Let's meet at Curlers” requires a shared understanding between the interlocutors about the place referred to through the toponym Curlers.^2^

By contrast, place‐like count nouns (definite object types) convey generic information about a place and its properties, and therefore relate to locale. Locale, in its broadest sense captures the properties of a place that are both perceptible and salient to some community. Accordingly, the notion of locale is tied to experience, affordance, and function. Tversky and Hemenway (1983), in their account of environmental scenes—which are places according to the derivation above—collected empirical data describing attributes, parts, and activities associated with such environmental scenes. They found that most listed terms were perceivable within a scene, rather than more abstract concepts, and that *parts* dominated the lists. Purves, Edwardes, and Wood (2011) found parts (elements) to be the richest vocabulary used to describe images of environments, followed by qualities (broadly equivalent to attributes) with activities used least commonly in the corpora of images analyzed. Nonetheless, many of those working on the semantics of place from a formal standpoint have emphasized the importance of activities, often from the perspective of affordances, in modeling place (for example, Jordan et al., 1998; Kuhn, 2001; Scheider, Janowicz, & Kuhn, 2009; Scheider & Kuhn, 2010).

The final element of Agnew's triad, *sense of place*, is central to notions of place. Sense of place is typically recognized as being related to the attachment a group of individuals has to a place (Cresswell, 2004), or the emotions people ascribe to a particular place. Sense of place is thus necessarily subjective, and varies according to culture and experience, not just of the place in question, but also with respect to its relations to other places (Cross, 2001). Different places can have the same affordances, such as resting (for example, bedroom, beach, park) or trading (for example, a market, or a mall), and any given place typically has a variety of affordances, sometimes differing for different communities. Thus, a beach may afford rest for some people, and for others, or at other times, play or even work. Going beyond Gibson's literal description of affordance but still aligned with his intentions, we explicitly include the affordance *to feel* in a particular way, which is a major contributor to sense of place. For example, people associate in an unreflected, subconscious manner a dark environment with fear (Kahneman, 2011). Others refer to *function* as place‐making (Papadakis, Resch, & Blaschke, 2016), which is less individual, but pointing in the same direction. Attached to this sense of place is a perception of *gestalt*, or wholeness (Metzger, 1931; B. Smith, 1988; Wertheimer, 1925), or continuity and distinctiveness (Twigger‐Ross & Uzzell, 1996). This gestalt can be grounded in a thing itself, in a particular configuration of things, or in a visual representation of things, where in each case the wholeness is inherited from the perceived contrast with other places (Winter & Freksa, 2012). Thus, Soho in London is an identifiable place, with well‐known properties (for example, theaters, music, and restaurants) which contribute to this sense of wholeness, and contrast it with the nearby shopping areas of Oxford Street. Any of these contributors to a sense of place add to the notion that places are objects, with properties.

Concluding this analysis of Agnew's three dimensions of place, places have some shared properties, even if the perception of these properties is not universal, may be bound to culture or language, and need not be permanent. It is well known that cultures or languages form geographic categories differently (Mark, Smith, & Tversky, 1999; Mark & Turk, 2003). Our analysis uses examples in English, and care is required not to assume that these examples are the only, or even an appropriate way of modeling place in other languages and non‐Western cultures. Indeed, the difficulty of translating place (as a word, and as a concept), even to other European languages, suggests that significant differences exist. Nonetheless, given the dominance of English in information systems, we suggest that notions that recognize the complex nature of place, but make it computationally tractable, are useful and may provide a fruitful basis for further cross‐cultural and cross‐linguistic studies.

In this section we have shown how our derivation of places (including the potential of places to form networks and participate in events) is compatible with the dimensions of place identified in the literature. Thus, we can now move on to computational aspects and highlight with concrete examples the compatibility of our approach with recent work on extracting place knowledge for information systems.

## Capturing, Managing, and Analyzing Place Data in Information Systems

Because we are concerned with the conceptualization, capture, management, and analysis of geographic information, and our proposal is a possible conceptualization for place information, we now illustrate the use of our conceptualization in producing and using actual information systems. We do so through three examples illustrating different use cases for place information.

In the first two of these, we explore the naming of places from contrasting perspectives: (a) a pan‐European government specification for the management of geographic names, and thus focusing on metadata, and (b) a range of attempts to capture information about geographic names from online sources. Our third example (c) moves away from place names and looks at ways of capturing the properties of places allowing their characterization and comparison. In a practical sense, our analysis also reveals important fields to be populated if we are to develop metadata specifications for place information.

Because we argue that place knowledge is a form of abstraction from reality rooted in shared experience, that is, based on human perception and cognition rather than technical measurements, the most obvious access to such knowledge is capturing ways in which people externalize knowledge about place.

**Figure 1:**
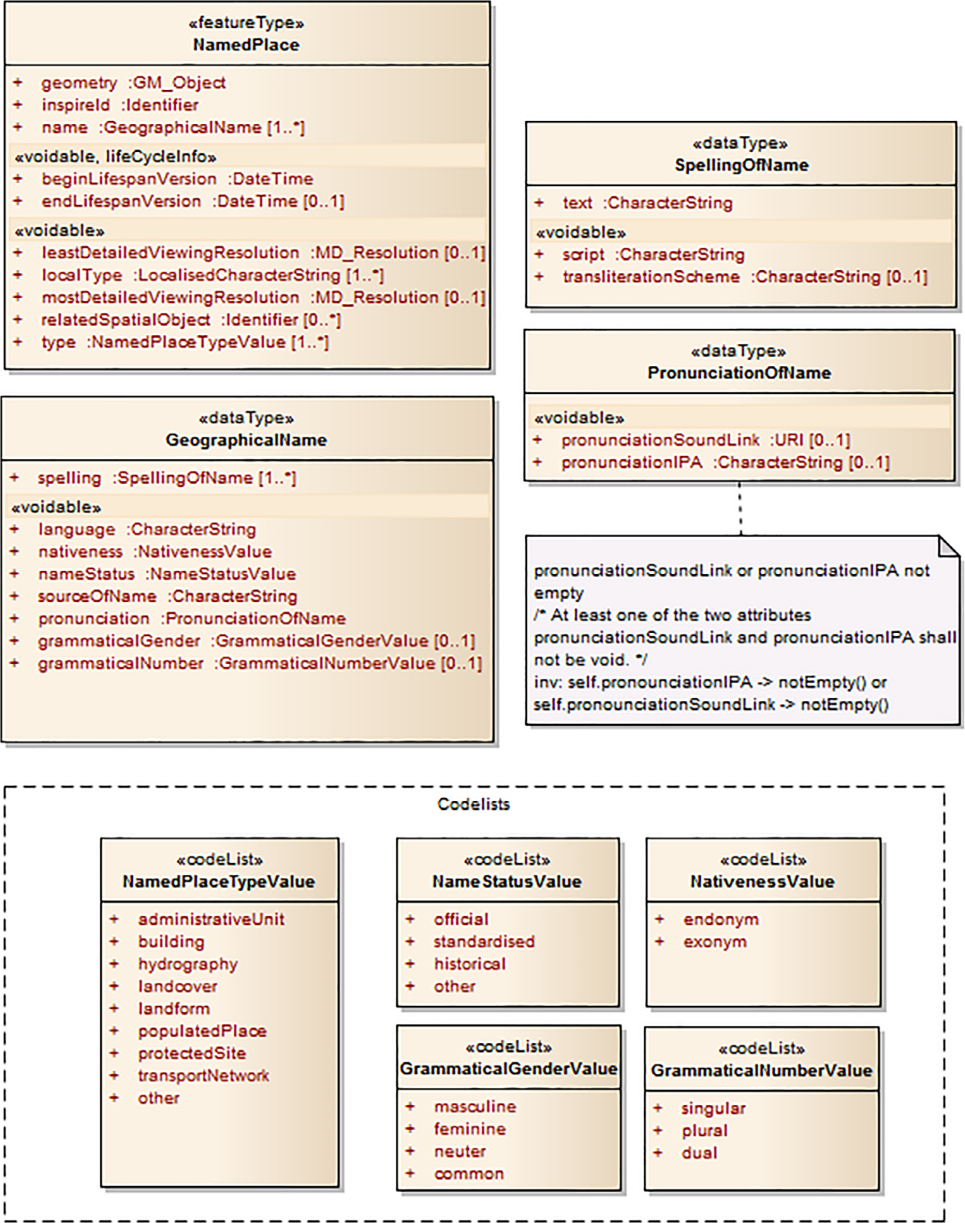
UML diagram illustrating the complexity of managing place names in the INSPIRE data specification (source: https://inspire.ec.europa.eu/documents/Data_Specifications/INSPIRE_DataSpecification_GN_v3.1.pdf). [Color figure can be viewed at http://wileyonlinelibrary.com]

One obvious example of such externalizations is the way in which information systems deal with place names. Historically, the maintenance of lists of authoritative place names was important both politically and for purposes of inventory (Hill, 2006). The INSPIRE data specification on geographical names (INSPIRE, 2014) recognizes this importance, and also implicitly illustrates the complexity of dealing with what at first glance appears to be a simple requirement of linking names to geometries (Figure 1). This complexity emerges from the need, among other issues, to deal with geographic names for places across national borders, including multiple languages and writing systems, and to take account of changes in time. The INSPIRE standard focuses on the management of named places defined as “real world entities referred to by one or several proper nouns” (p. 15) explicitly aiming to allow existing data to be represented and linked. At its heart lie names, which take the role of object identifiers in the core concepts. All other properties are related to a place through its shared identification, and are thus captured in an object associated with a location. For example, the location associated with the name London reflects some shared spatial conceptualization of this place at some particular time. Modeling a place as an object with a location, allows for a range of calculations. For instance, we can query for all the names by which London is known, identify places contained by London (using spatial reasoning), or find all places with the same type as London. However, the characterization of places in this standard is limited, and an underlying assumption is that a given named place is associated with a single (potentially compound) geometry and may or may not be in contemporary usage. Furthermore, the INSPIRE specification focuses on the “description of names rather than the description of spatial objects” (p. 1).

A recognition that locations might be associated with different places, and that changes in the name used for a place might also imply a different shared identity for a place, are among the questions motivating work on the capture and management of vernacular or informal place names. A pragmatic need, for example, in linking web resources to place names, and the emergence of large volumes of data on the web containing place names, both as metadata and in natural language, has led to the extension of methods concerned with extracting place names and their geometries through in situ questionnaires (for example, Montello, Goodchild, Gottsegen, & Fohl, 2003). Data‐driven elicitation methods using place names in natural language and tags from social media essentially rely on spatial autocorrelation and spatial relationships in delineating regions associated with informal place names. In terms of core concepts, it is possible to conceptualize a field representing the degree to which any location belongs to, say, downtown Santa Barbara (cf. Gao et al., 2017; Grothe & Schaab, 2009; Hollenstein & Purves, 2010; Jones, Purves, Clough, & Joho, 2008). We can then identify locations having a greater likelihood of being part of the place object downtown Santa Barbara. This object can also be, for example, part of south California. Thus, in contrast to our previous example, here the name of a place is a property of some object. Using such representations allows us to query, for instance, locations in London that are more or less characteristic of London as conceptualized by a particular group (for example, as a shared conceptualization in a language or discourse). Such analysis possibilities capture notions of instances of places as shared but not universal conceptualizations within a single data model.

Our first two examples focused on place names, or as suggested by Agnew, the notion of location (Agnew, 2011). Locale is reduced to place types in such data models, and sense of place emerges from associations individuals might have with instances of place names and types. In our last example, we turn to capturing, managing, and analyzing place properties going beyond names, focusing on the use of online sources. Perhaps the most obvious sources of such knowledge are actively crowd‐sourced data, either with explicit or implicit geometry such as OpenStreetMap and Wikipedia. OpenStreetMap elicits place knowledge in the form of geometry and its properties—for instance, mapping roads and paths. This geometry is produced by volunteers, and therefore perhaps nearer to conceptualizations of place than traditional top‐down spatial data. However, the need to produce a coherent map representation means that in practice OpenStreetMap, although containing information about physical properties of space relevant to locale, is little more nuanced than traditional, authoritative spatial data (although perhaps more subject to biases that implicitly reflect some properties of place; Haklay, 2010; Quattrone, Capra, & de Meo, 2015). Georeferenced pages describing locations in Wikipedia are also a rich source of place knowledge (Overell, Sigurbjörnsson, & Van Zwol, 2009), although with similar challenges of bias. Graham, Hogan, Straumann, and Medhat (2014) found that Wikipedia “remains characterized by uneven and clustered geographies”—in other words, many places are not captured in such databases.

As well as using actively crowdsourced data, a number of approaches to generating place information have sought to mine social media and text. Methods essentially either rely on georeferences explicitly stored in the form of coordinates, or first identifying and disambiguating geographic referents, such that they can usefully be located in a place database. Whichever approach is taken, there are essentially two ways of managing the data extracted. First, place properties can be linked to place names through triplets, as popularized in linked data models, and thus be considered as properties of named objects (Kim, Vasardani & Winter, 2016). Second, quasi‐continuous surfaces based around, for instance, emerging terms from topic modeling (Adams & McKenzie, 2013; Brown, Baldridge, Esteva, & Xu, 2012) or lists of landscape terms extracted from a corpora (Derungs & Purves, 2014) are captured. By querying within a given region, it is then possible to identify other locations associated with similar properties. Thus, one might query data in Switzerland for locations described through mountain‐like properties (for example, peak, glacier, steep, high, rough, and so on) and identify regions associated with “mountainness” (cf. Derungs & Purves, 2016).

Social media data have received particularly widespread attention as a source of more nuanced, subjective place‐information (Crampton et al., 2013). Thus, georeferenced Twitter data have been used to explore how individuals experience a city spatially, and thus give insights into a subjective sense of place (Mitchell, Frank, Harris, Dodds, & Danforth, 2013; Shelton, Poorthuis, & Zook, 2015), whereas sentiment analysis on georeferenced Flickr images has been used to extract a two‐dimensional representation of emotions based on valence and arousal (Hauthal & Burghardt, 2016). These maps represent fields from which places as located objects can be derived, often with changing properties over time.

In terms of the core concepts and place properties, it is once again clear that there are essentially two ways of thinking about (and thus managing and analyzing) place data. Either properties are associated with existing places (and therefore preexisting locations and associated objects) OR properties are stored as fields or networks and can be queried to identify new regions, which may or may not then become places. Thus, for example, all the regions described as steep simply capture a property of landscape, but not a particular place or group of places. But, all the steep, rocky and high places in Scotland might very well describe a region which is considered Scottish wilderness—and this region does share many of the properties of a place.

## Conclusion and Future Directions

The concept of place continues to be the subject of extensive research in geography, as well as in the social and environmental sciences (for example, Raymond, Kyttä, & Stedman, 2017). In geography, a desire to deal with places computationally and to develop so‐called platial systems, has resulted in a flood of literature in recent years. By contrast, in computer science and information science, place remains an ill‐defined attribute of database records, rather than a clearly defined and useful concept.

Our key contribution in this article is to clarify the notion of place from the perspective of its representation in information systems. In particular, we demonstrated the links between, on the one hand, an ontology of spatial information, and on the other, the social and cognitive properties of places articulated in the literature. In so doing we link a formal, theoretical model of core concepts to theory about the nature of place, and show how these two viewpoints are reconcilable. Furthermore, we go a step further than most previous work, and show how existing efforts to extract individual properties of places can be aligned with our model and, more important, how using this model allows us to derive a range of possible computations from place data.

We propose, based on core concepts of spatial information, the following ontological commitment for place definitions:

A *place* is an object resulting from a shared identification of a location. As an object, it may become a part of a network and participate in events.

By making an ontological commitment, and in particular specifying the three conditions associated with this commitment, we establish a language through which we can productively work with many existing definitions about places and move toward a shared understanding of the general properties expected from information systems dealing with place. Furthermore, our ontological commitment is specific enough to ensure that definitions of place must meet a basic set of shared conditions.

This commitment has a number of implications for information systems:

1. Places are central to human communication, and are thus essential concepts for any information systems dealing with *where*‐type questions.
2. Places are not adequately represented by either geometries or names alone because these are insufficient to define both an object and a location.
3. Object properties and relations allow us to represent places, without a requirement for fixed boundaries, while still having them bounded in space and time.
4. Formalizing and supporting richer notions of place is possible by extending existing data models and metadata specifications, and will result in information systems that better meet user needs.

Based around these implications we see multiple possibilities for further work. We argue that our proposal suggests ways of explicitly including and enriching the modeling of place conceptually. Doing so would have significant benefits for retrieval of information from the resulting data structures, enabling, for example, a clearer distinction between *what* and *where* questions through a clear separation of objects and their properties from locations. Furthermore, the adopted spatial information ontology suggests studying how the core quality concepts of spatial information (granularity, accuracy, and provenance) guide quality assessments of place information—an aspect outside the scope of this article. Finally, by treating place as a shared conceptualization, its importance as a form of context becomes clearer and its incorporation in both retrieval and analysis more straightforward. Explicitly considering place as a form of context, and not, as presciently pointed out by Harrison and Dourish (1996), ignoring the distinction between space and place, should lead to information systems that better match real‐world information needs.

## Endnotes

1
https://www.fgdc.gov/initiatives/framework/

2 Curlers is a well‐known bar in the west‐end of Glasgow, where one of the authors spent many happy hours during his studies.
